# The status of imported Barremian-Bedoulian flint in north-eastern Iberia during the Middle Neolithic. Insights from the variscite mines of Gavà (Barcelona)

**DOI:** 10.1371/journal.pone.0224238

**Published:** 2019-11-06

**Authors:** Ferran Borrell, Josep Bosch, Juan Francisco Gibaja, Patrick Schmidt, Xavier Terradas

**Affiliations:** 1 Spanish National Research Council (IMF-CSIC), Barcelona, Spain; 2 Museu de Gavà, Gavà, Spain; 3 Department of Early Prehistory and Quaternary Ecology, Eberhard Karls University of Tübingen, Tübingen, Germany; University at Buffalo - The State University of New York, UNITED STATES

## Abstract

Barremian-Bedoulian flint from the Vaucluse region (western Provence, SE France), is traditionally considered one of the most significant chrono-cultural markers of the Chasséen culture during the Middle Neolithic (end of the 5^th^ and beginning of the 4^th^ millennium BC). Diffusion of Provençal flints became massive during the first half of the 4^th^ millennium BC, penetrating in several neighbouring cultural spheres such as the Sepulcros de Fosa culture in north-eastern Iberia. The integrated study of the lithic assemblages from the variscite mines of Gavà (Barcelona) and its contextualization within the Sepulcros de Fosa culture in north-eastern Iberia have revealed unexpected complexity in the modes of consumption, use and status of imported Barremian-Bedoulian industries in north-eastern Iberia during the 5^th^ to 4^th^ millennia cal. BC transition. Local communities within this region, already controlling extraction and regional diffusion of variscite ornaments, exerted control over the fluxes of Vauclusian flint south of the Pyrenees, where it had a triple status (functional, symbolic and both). In addition, the results provide complementary data to better understand relevant aspects of the nature and organisation of Barremian-Bedoulian flint exploitation and early supply systems at the Provençal producing sites during the later phase of the Chasséen culture.

## 1. Introduction

The Neolithic transition in the north-west of the Mediterranean Basin (5500–3500 cal. BC) is characterised by a remarkable shift in the complexity and intensity of inter-regional social interaction and trade networks, particularly those involving abiotic resources (e.g., obsidian, flint, Alpine rocks, steatite, variscite, etc.) [1–5]. A paradigmatic example is found in southern France during the Middle Neolithic (end of the 5^th^ and beginning of the 4^th^ millennium BC), when lithic industries relied on the large-scale production, use and diffusion of Barremian-Bedoulian flint from lower Aptian outcrops in the Vaucluse region (western Provence, SE France), which become one of the most, if not the most, significant chrono-cultural markers of the *Chasséen* culture. A series of long-term technological and functional studies focused on these lithic elements has enabled not only the characterization of the *Chasséen* culture lithic productions and modes of diffusion, but also a better understanding of the territorial organisation of the *Chasséen* culture communities [6–14]. In this sense, different authors agree that the massive and specialized exploitation of this raw material and distribution of the products (and obsidian to a lesser degree) was the main vector through which settlements were articulated and hierarchized: workshops near the outcrops, knapping settlements (use and early distribution), consumption and redistribution settlements and, finally, consumers. Accordingly, in the same line of interpretation, the *Chasséen* culture can be defined through the social complexity that developed in the framework of a producing-consuming settlement system, the presence of specialized productions and highly complex technical processes (heat treatment and pressure *débitage*) and, finally, the development of extensive and intense exchange/interaction networks.

**Fig 1:**
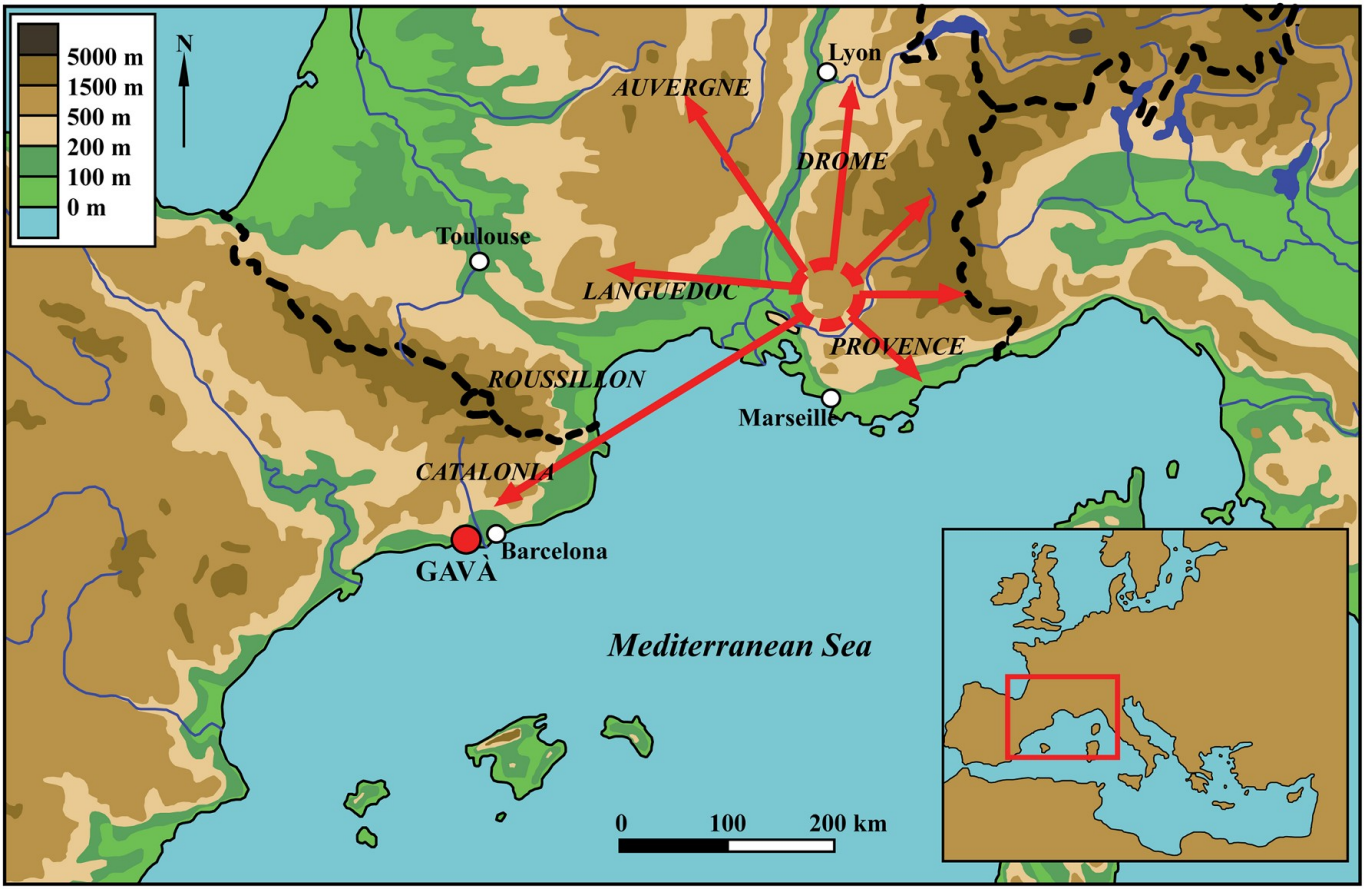
Source area and diffusion of Barremian-Bedoulian flint in the north-western Mediterranean basin (modified from ref. [12]).

It is broadly accepted that Barremian-Bedoulian flint from Provence, also referred to in the literature with alternative terms such as *silex blond*, *silex bédoulien*, *silex melado*, *silex melat* or honey flint, was distributed over a wide area of the south of France and beyond (Fig 1), though it should be noted that specific provenance analyses are lacking in some regions, such as north-eastern Iberia (see section 3 for details). Diffusion became massive between 4200/4000 cal. BC and the first half of the 4^th^ millennium BC, when blades/lets and cores penetrated in several neighbouring cultural spheres (e.g., *Sepulcros de Fosa* culture in north-eastern Iberia and *Vasi a Bocca Quadrata* and Lagozza cultures in northern Italy) [4,7,9–12,15–18].

Interestingly, the circulation of Barremian-Bedoulian flint around the *Chasséen* culture territories (Provence and Languedoc) and beyond was, however, not homogeneous, and different modes of consumption and use of Barremian-Bedoulian products have been identified. On the one hand, minor differences have been identified between the several groups or facies within the core *Chasséen* cultural area (e.g., Plots, Bize, Auriac, Cavanac-Toronde), where regional and local flints often formed a significant part of the assemblages [10]. For instance, in the Provence, the Rhône valley or the eastern Languedoc, the lithic productions of the Vaucluse are almost totally absent from grave goods and they were confined to technical uses. On the other hand, in the margins of the massive diffusion area, Barremian-Bedoulian blade products are also present in burial contexts (e.g., tombs in the Aude and in the Garonne valley) [19], thus identifying a double status of Barremian-Bedoulian productions: basic element of the technical system, integrated into everyday life, and highly-valued precious goods [10,11,16]. This change of status of imported productions in margin regions of the *Chasséen* cultural area is clearly related to the distance from the production zone but the reasons for that phenomenon are not clear/resolved yet, but merely geographical or cultural (due to crossing a cultural border).

A similar phenomenon has been identified in north-eastern Iberia, a region with a particular and biased archaeological record for the Middle Neolithic (large number of burial sites and very limited evidence of residential settlements), which entails additional interpretative difficulties when compared to southern France. In that region, blades, bladelets, projectiles (both points and geometrics) and cores made of Barremian-Bedoulian flint are commonly found as grave goods in the abundant funerary record of the region (Bòbila d’en Joca, Bòbila Padró, Bòbila Madurell-Can Gambús I, etc.), thus undoubtedly indicating that imported Barremian-Bedoulian productions had acquired a new status and use. However, early technological and functional studies revealed that the blade/lets and projectiles found in funerary contexts had often been used and, secondly, that the few Barremian-Bedoulian flint assemblages found in the scarce number of Middle Neolithic structures from residential settlements (e.g., Bòbila Madurell) included other tools not found in the funerary contexts, such as burins, scrapers and some elements (e.g., tablets, chipped debris, flakes, etc.) that might result from the knapping sequence. In light of this evidence, the possibility was suggested that Barremian-Bedoulian flint in NE Iberia had, as in the Languedoc, a dual status (functional and symbolic) and, secondly, that it might have been knapped *in situ* (at least in some cases and only partially [preforms of cores were always prepared at the producing sites in the Provence]) by local consumers [20,21]. However, characterization of the complex use and value of imported Barremian-Bedoulian flint in northeast Iberia needs further fine-tuning as interpretations are based on limited data which, in some cases, indicate a rather complex and varied picture.

This paper is aimed at refining our understanding of the use and value of Barremian-Bedoulian flint in NE Iberia and, accordingly, at providing further evidence of the status of imported Provençal flint in this region. The study presents the results of a comprehensive approach to the lithic assemblages from the variscite mines at Gavà (Barcelona, Spain) dated between 4200–3600 cal. BC, which constitute one of the very few non-funerary Middle Neolithic sites in NE Iberia, even though some mines were re-used as funerary places by the same community of miners [5]. The presence of Barremian-Bedoulian and other flint types in a range of contemporary but different contexts (burials in mines 83 and 84, a non-funerary deposit in mine 85 and habitat debris in mines 5/11 and 16) offers an excellent opportunity to reconstruct the distribution, use and status of imported Barremian-Bedoulian productions at the core of the *Sepulcros de Fosa* culture area in north-eastern Iberia during the Middle Neolithic. In addition, evidence from the distant consuming sites also enables a better understanding of the nature and organisation of Barremian-Bedoulian productions—the supply systems—performed at the core of the *Chasséen* culture area and, secondly, of the nature and functioning of exchange networks through which Barremian-Bedoulian flint circulated through the Western Mediterranean basin during the 5^th^ to 4^th^ millennia transition.

## 2. The site: Neolithic variscite mines of Gavà

The Neolithic mines at Gavà form a unique subterranean mining complex where variscite (a green phosphate mineral, similar to turquoise, also known as *callaïs* or *cal·laita*), was extracted and processed into ornaments which were often devoted to prestige goods (beads and pendants) by the miners themselves [5,22–25]. No direct evidence of the settlement associated with the mining structures has been found to date, even though it must have existed nearby as indicated by the range of finds (e.g., faunal remains, pottery sherds, lithic artefacts, daub fragments with impressions of branches, etc.) recovered in the fill of some of the mines.

Variscite ornaments made at Gavà have been found in the mines themselves (in the fills of the subterranean shafts and in the burials located deep inside some of the mines) and in many Neolithic burials in the north-easternmost part of the Iberian Peninsula and part of southern France during the Middle Neolithic. These valuable ornaments circulated, together with other lithic materials (e.g., Barremian-Bedoulian flint from south-eastern France, obsidian from Mediterranean islands [2,3] and jadeite and other Alpine rocks [1]), through complex and overlapping trade networks, bringing into contact different contemporary Neolithic cultures in the north-western Mediterranean basin.

Mining activities at Gavà were concentrated in the first half of the 4^th^ millennium cal. BC, although slightly earlier and later activity is also reported. Mine structures vary greatly in complexity and size, from short, simple, vertical or semi-vertical shafts to complex structures that combine semi-vertical shafts, distribution chambers and horizontal tunnel-like galleries at different levels, reaching a total depth of up to 15 m. The surface area affected by the mining activities was quite large, probably several hectares. A dozen mines have been fully or partially excavated, while over 90 sections of shafts or galleries have been identified and left unexcavated for future research.

**Fig 2:**
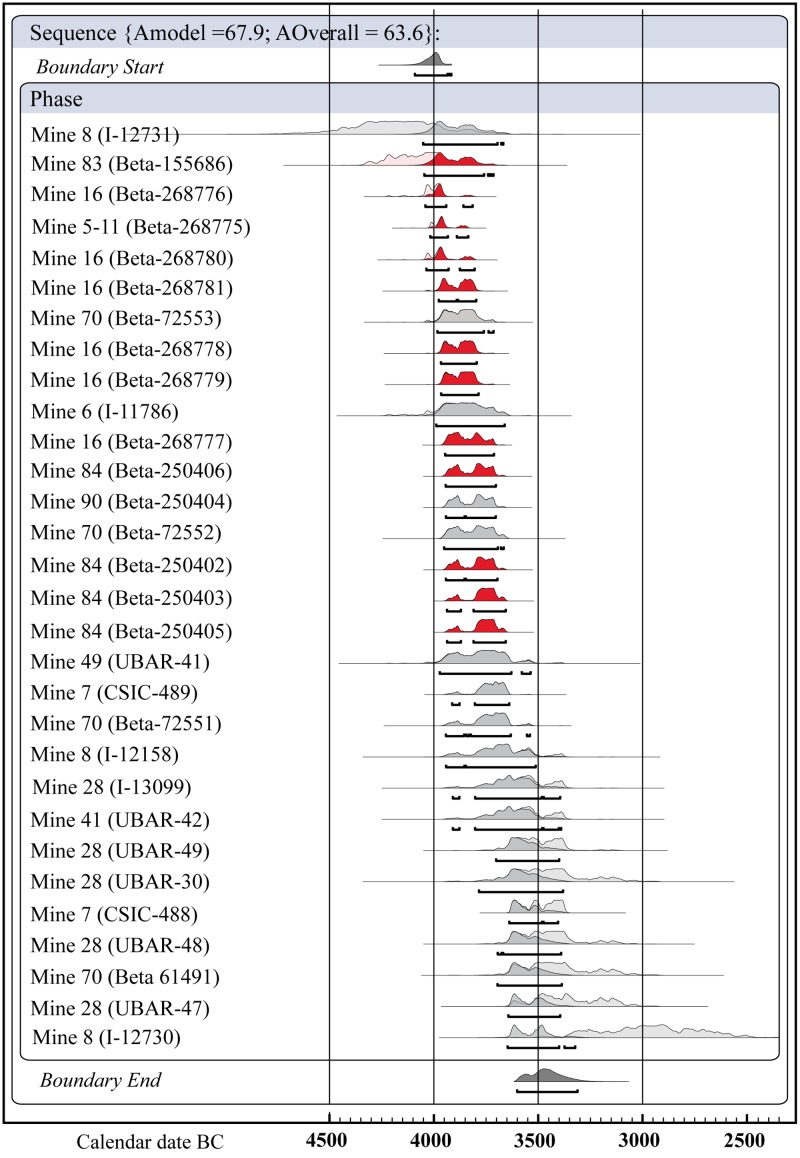
Results of a single phase Bayesian model applied to ^14^C dates from the Neolithic variscite mines. The 14C dates from the mines the lithic assemblages presented in this paper belong to are shown in red. Generated in OxCal v4.2.2. [26] using IntCal13 calibration curve [27].

**Fig 3:**
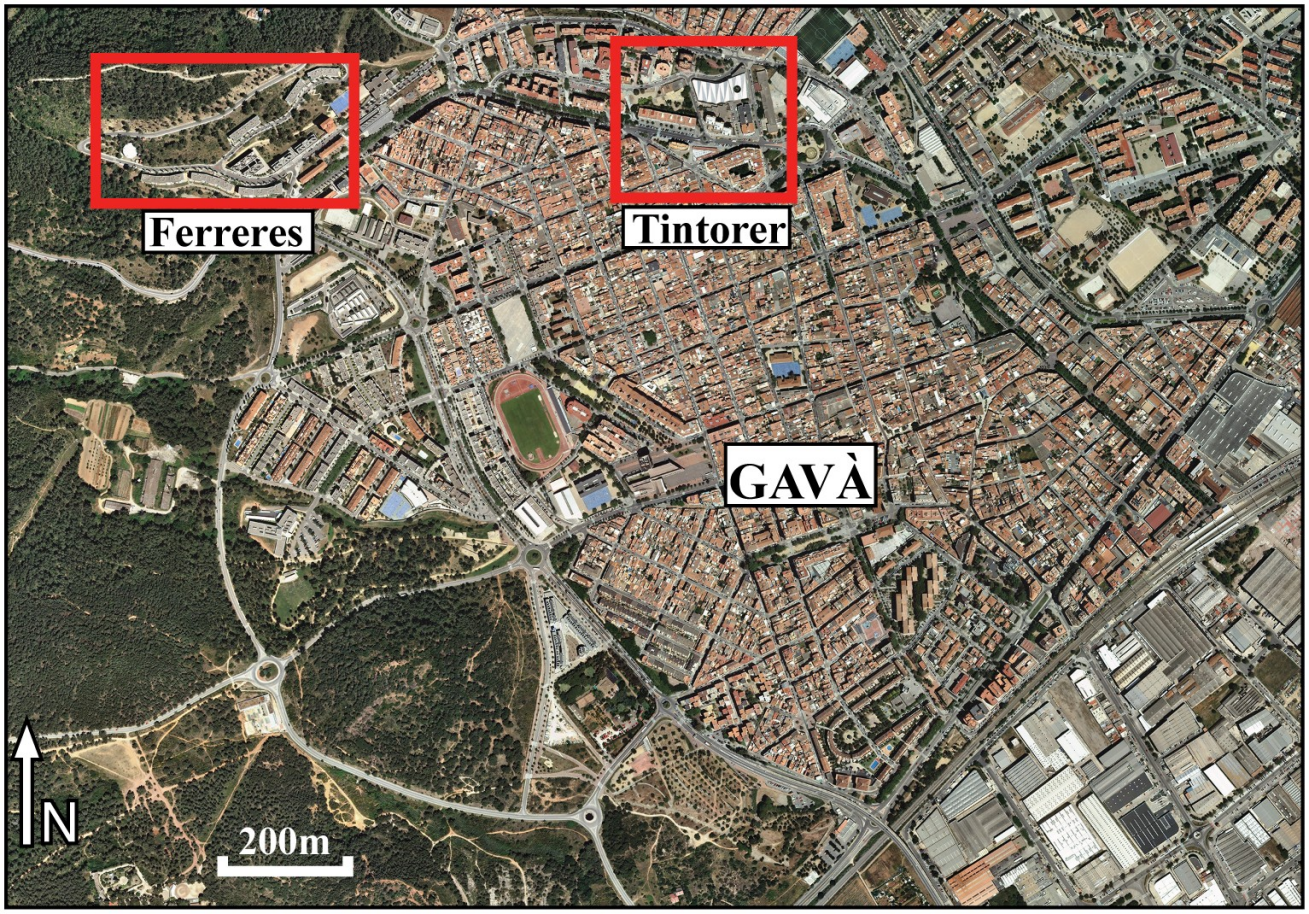
Orthophoto of Gavà at 1: 2,500 with the location of the two mining sectors mentioned in the text. Source: Institut Cartogràfic i Geològic de Catalunya (ICGC), under a CC BY 4.0 license.

The lithic assemblages studied comprehend a range of contexts from different mines in two sectors, though they are all strictly contemporary (Figs 2 and 3). The study of the lithic assemblages (339 items) has been carried out in collaboration and with the permission of the Museu de Gavà (Barcelona, Spain), where the artefacts are stored, some of them as part of the permanent exhibition, but they are publicly accessible to other researchers.

Mines 83 and 84 are located in the Ferreres sector (41°18´31.69´´N, 1°59´26.77´´E) and have been completely excavated. Mine 83 is a small simple mine that only reached a depth of 4m, while Mine 84 is a medium-sized (10×14 m) T-shaped mine, combining narrow galleries (1 m in diameter) with wider chambers (6.5 m deep). Both mines were re-used as burial sites during the peak mining period at Gavà to bury the bodies of one and two individuals, respectively, who have been interpreted as miners [5]. Grave goods accompanying the bodies were abundant; including Barremian-Bedoulian flint, variscite beads, an obsidian blade, eclogite axes, a square-mouth vessel, etc. The small flint assemblage from both mines was found in the funerary chambers, which were closed by middle-sized stone slabs, while the rest of the mines were filled and sealed with ‘clean’ mining debris with extremely limited archaeological material (fragments of picks and a few potsherds). Of Mine 85, only two distal ends of semi-vertical shafts were preserved and, thus, excavated, reaching a total depth of 4 m. The lower shaft was filled with organic clay/silt-based anthropogenic deposits with a few faunal remains and shells. The upper shaft, filled with clean mining debris contained an extremely rich deposit that included three complete pottery vessels, abundant variscite items (beads, perforated plaquettes, pendants and medals), several bone tools, flint blades and arrowheads and other objects.

Mines 5–11 and 16 are located in the Tintorer sector (41°18´35.90´´N, 1°59´59.97´´E). Mine 5–11 corresponds to the largest mining structure of the Gavà Neolithic complex and it has only been partly excavated [28]. The complexity of the structure is remarkable, as it combines a series of shafts and chambers at different levels and reaches a total depth of 15 m. The mine was filled with mining debris, thus archaeological artefacts were very scarce, mostly a few potsherds. Fragments of picks and very few flint artefacts were recovered. In the same sector, part of Mine 16 was excavated. It corresponds to the semi-vertical entrance shaft of a mine that reached a depth of almost 9 m, thus suggesting that it was a medium- to large-sized complex mining structure. The shaft was filled with clay/silt-based anthropogenic deposits that included relatively abundant archaeological artefacts (lithics, pottery sherds, faunal remains, marine shells, charcoals, seeds, picks, fragments of variscite beads, daub fragments with negative impressions, etc.) and the anthropomorphic figure-vessel known as the Gavà Venus [5,29].

Although the core data for this study are based on the most recently excavated mines at Gavà, our analysis and interpretations also integrate all data about the lithic assemblages recovered during the excavations undertaken during the early 1990s (Mines 65, 68, 69, 70 and 71), where the presence of imported Barremian-Bedoulian flint was reported [30], as well as old and new data from Middle Neolithic sites in the north-eastern Iberian Peninsula.

## 3. Barremian-Bedoulian flint at Gavà mines

Detailed mineralogical, chemical and petrographic characterization of Barremian-Bedoulian flint from south-eastern France has been undertaken recently, concluding that despite the remarkable intra-formation variability observed [31], a series of features and elements can be considered as ‘markers’ that might help distinguishing Barremian-Bedoulian flints from other macroscopically similar (light brownish to yellowish in colour and with homogeneous texture) Turonian or Bajocian flints [32]. Like most flints and cherts, its matrix is made of chalcedony in small-unoriented domains [33]. Chalcedony is mainly present as length-fast chalcedony 85%, with a minor amount of length-slow chalcedony (15%) [34]. Up to 10 vol% of Opal-CT lepisheres and ~5 vol% of carbonate, resulting from incomplete silicification, have been observed within this chalcedony matrix, using thin section microscopy [35]. Surface observations using stereomicroscopes have described visible elements within the matrix that account for up to ~20% of the observed field of view: mainly detrital quartz grains and remains of sponge spicules [32]. Barremian-Bedoulian flint contains ‘water’ in the form of chemically bound water (SiOH) of ~56 wt% and as molecular water (H_2_O) in fluid inclusions of ~0.33 wt%; it encloses a total intergranular pore volume of ~0.65 vol% [36]. All these characteristics confer a good knapping quality to this type of flint, including good homogeneity in terms of force transmission, high fracture strength and low fracture toughness [37], and provide the possibility to heat-treat the rocks to improve knapping quality even further [38].

As mentioned in the introduction, no specific provenance studies have been undertaken in north-eastern Iberia, thus the Provençal origin of the fine-grained honey-coloured flints found south of the Pyrenees is yet to be corroborated with archaeometric criteria. Nevertheless, a series of features/evidences have led to the general agreement, by both French and Spanish researchers, that a Provençal origin of the fine-grained honey-coloured flint found in north-eastern Iberia during the Middle Neolithic is the most reasonable hypothesis in the current state of knowledge [39]. These features include, but are not only restricted to, 1) the absence of similar flint types showing the above-mentioned diagnostic petrological criteria, not only at a regional scale [40] but in the rest of the Iberian Peninsula [41]; 2) the distribution and chronological timespan honey-coloured flint is found in in north-eastern Iberia, in coincidence with the peak exploitation of Provençal sources, suggests a northern origin (beyond the Pyrenees) for this raw material and, finally 3) the great technological similarities (pressure flaked blade production) between the Provençal and Iberian assemblages. Accordingly, honey-coloured flint found in north-eastern Iberia has been incorporated as Barremian-Bedoulian in several studies about the vast exchange networks of exogenous products and raw materials taking place during the Middle Neolithic in the Western Mediterranian basin [10,11,39].

### 3.1. Mines 83, 84 and 85

**Fig 4:**
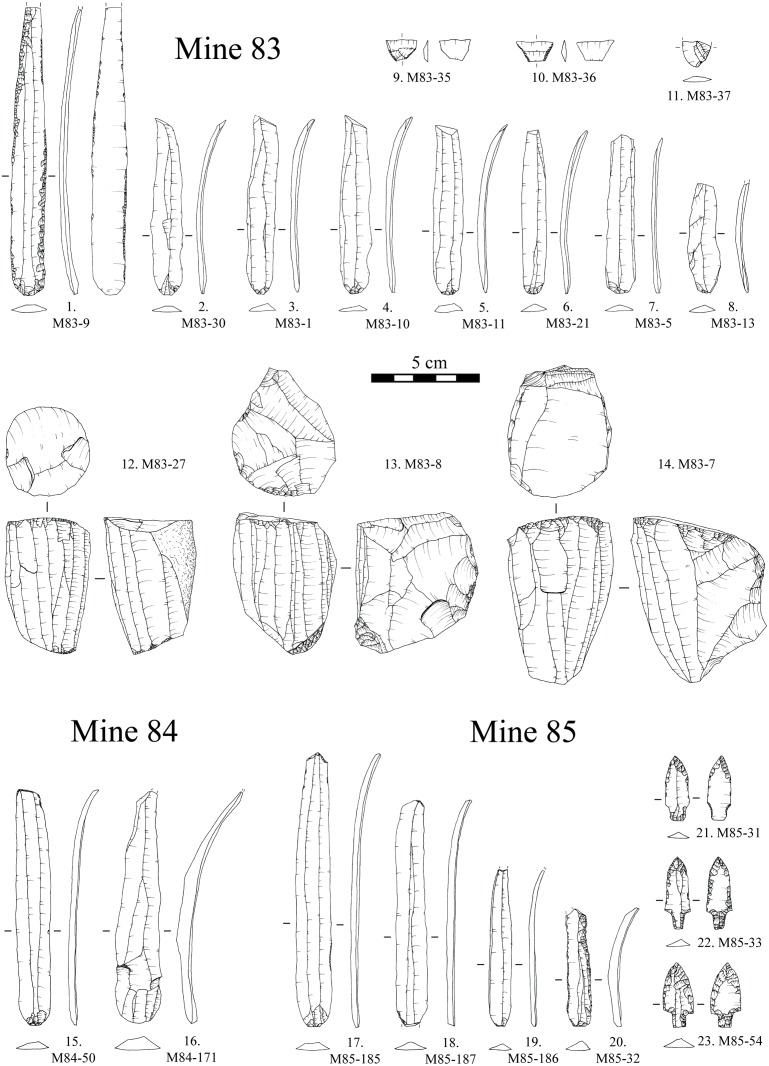
Flint assemblages from Mines 83, 84 and 85 in the Ferreres sector.

**Fig 5:**
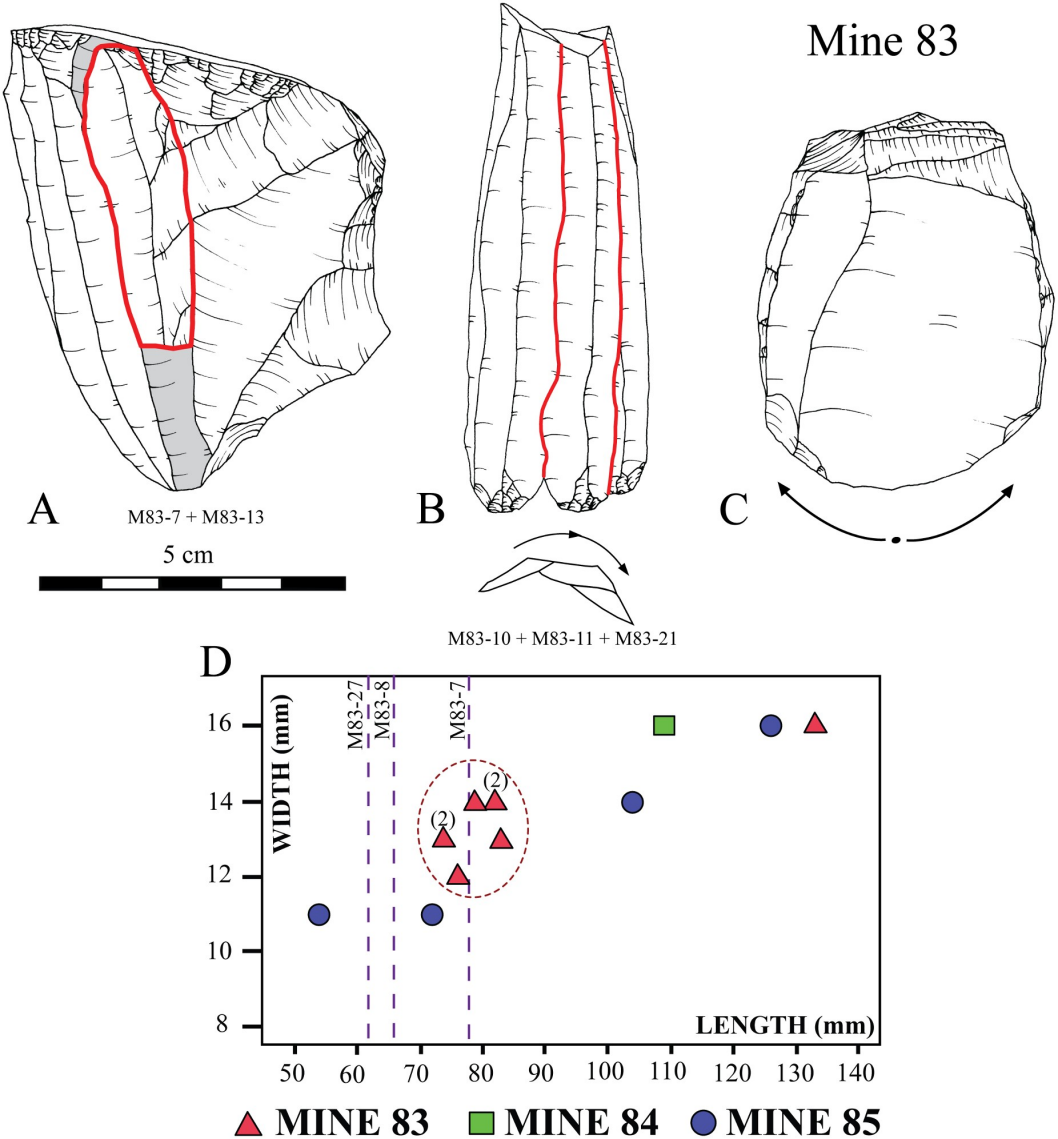
Refittings of Barremian-Bedoulian flint from Mine 83. Core and blade (A) and a set of three blades (B); direction of the knapping sequence in the same core (C); and measurements of the complete Barremian-Bedoulian flint blades from the Ferreres sector (D). Dotted lines in D indicate the maximum length of the exploitation surfaces of Provençal cores recovered in Mine 83.

The lithic assemblage recovered in funerary (Mines 83 and 84) and other ritual (Mine 85) contexts is formed by 3 cores, 3 arrowheads, 2 trapezes, 14 complete blades and a proximal fragment of a blade (Fig 4). All the artefacts were produced with Barremian-Bedoulian flint, except for a blade made with medium-grained local white flint (Fig 4: 16) and an arrowhead made of Lacustrine Oligocene banded flint from the Ebro Basin (Fig 4: 23). The assemblage includes the largest blade made of Provençal flint found in the NE Iberian Peninsula; up to 13.3 cm in length despite the loss of the tip (Fig 4: 1). The blade found in Mine 84 (Fig 4: 15) and two of the blades from 85 (Fig 4: 17, 18) are also among the largest blades of Barremian-Bedoulian flint found south of the Pyrenees, measuring 12.6, 10.9 and 10.4 cm in length respectively. Among the series of medium-sized blades from Mine 83, three have been refitted and another one (missing its distal part) refits with one of the cores found (Fig 5A, 5B and 5D). Observation of the morphological features (regularity, almost straight profile, thin section, small platform, etc.) and measurements, and macroscopic features of the raw material used of all the medium-sized blades from Mine 83 (Fig 4: 2 to 8) suggest that they all correspond to the same knapping sequence of one of the cores (Fig 4: 14), though some blanks are missing. The pressure technique was used to knap both the large and medium-sized blades made with Barremian-Bedoulian flint, except for the shortest one found in Mine 85 (Fig 4: 20) which, together with the large blade made with white flint (Fig 4: 16), display very different technological features (thick, very curved, without parallel edges and negative dorsal removals, marked bulb, etc.). In one case, at least, it has been possible to determine that the knapping sequence started from the middle of the knapping surface towards the laterals of the core (Fig 5C) [42]. The distribution of the width and length values of the blades produced by pressure reveals a cluster of products with values between 11 and 14 mm wide and no longer than 9 cm, and a few products (the larger blades) ranging between 14 and 16 mm wide and between 10 and 14 cm in length. Comparison of the width ranges with experimental studies oriented to discriminating different modes of pressure microblade/bladelet/blade production [Pelegrin 2012] revealed the use of the standing pressure technique (mode 4) for the production of medium- to large-sized blades made with Barremian-Bedoulian flint. Interestingly, four of the medium-sized blades (Fig 4: 2–5) display an s-shaped profile on one of the edges and/or the dorsal negative removals, suggesting a problem of stability of the core (e.g., non-adequate holding) that was finally solved (Fig 4: 6).

The blade assemblage from Mines 83, 84 and 85 displays little retouch except for the large blade (Fig 4: 1), with abundant direct and inverse retouch on both edges, and the shortest curved one (Fig 4: 20), displaying abundant direct retouch on the right edge and marginal, also direct, on the left. The other large blades, from Mines 84 and 85, have minimal direct retouch concentrated on the distal and/or proximal ends. The rest of the Barremian-Bedoulian blades (medium-sized blades corresponding to the same knapping sequence of one of the cores) and the blade made with local white flint display no evidence of modification.

All the flint artefacts from Mines 83, 84 and 85 were also studied for use-wear analysis using a KYOWA TR-P binocular (10X-90X) and an Olympus DH2-UMA metallographic (100X-400X) microscope, while photographs have been taken with a Canon 450D camera using Helicon Focus v. 4.62 software. For the functional interpretation, several macro- and microwear traces were evaluated: scars, rounding, striations and micropolish. Such traces are diagnostic for determining both the motion of tool utilization (kinematics) and the worked material, as well as for detecting surface alterations [43–48]. The functional diagnosis was established by comparing the archaeological samples with the experimental reference collection of the IMF-CSIC in Barcelona, Spain. The collection is the result of many years of research and experimental analyses conducted by the members of the Archaeology of Social Dynamics research group [20,49–51].

**Fig 6:**
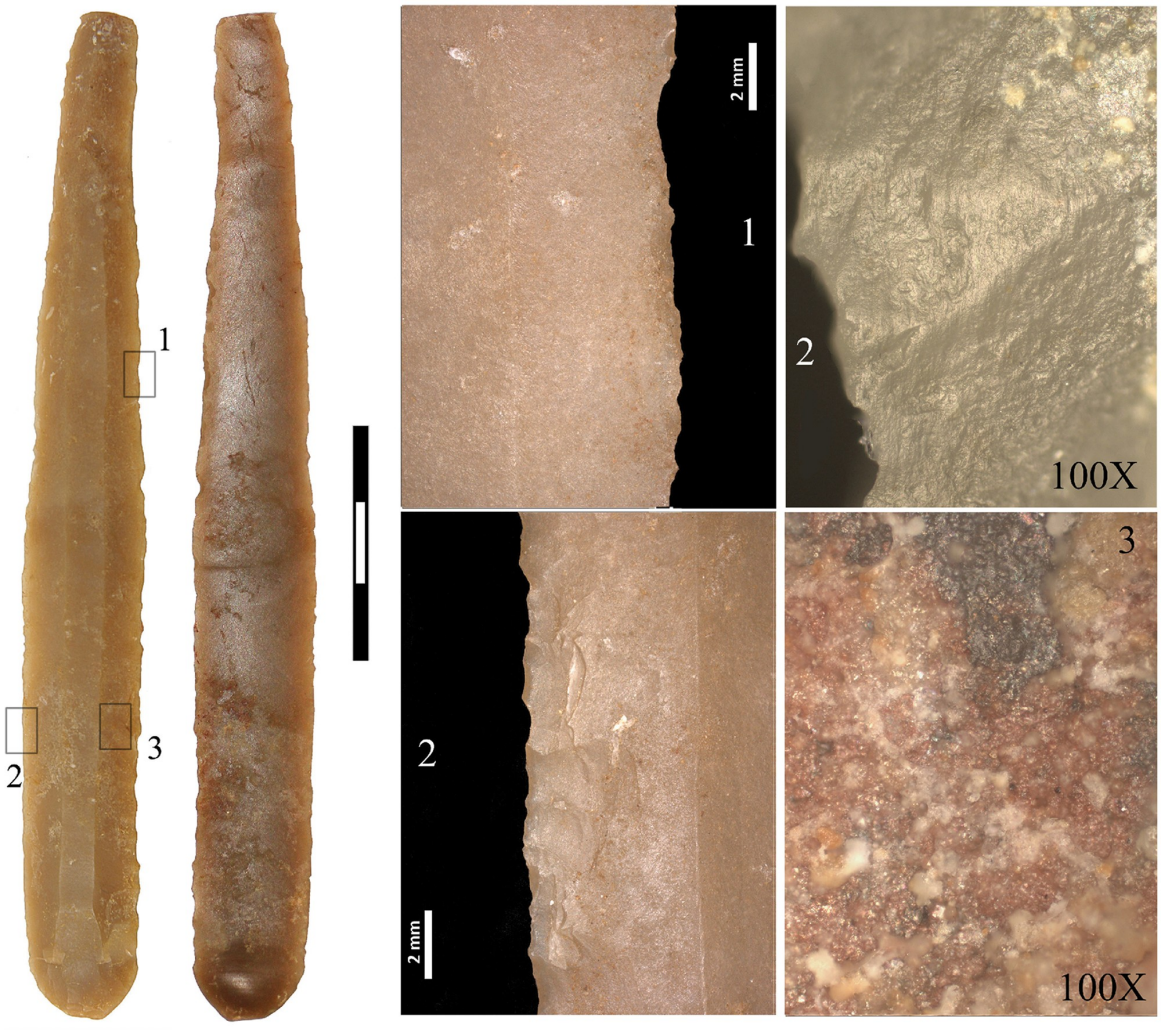
Diagnostic use-wear of soft-plant processing on both edges of the largest Barremian-Bedoulian blade found south of the Pyrenees. Note that both edges are retouched (1) and at microscopic level display marked rounding and abrasion that extends towards the centre of the blade. The polish does not look very compact because of the numerous striations characterising the abrasion (2). Blackish residue in the central part is possibly a product of the material used to haft the blade (3).

Diagnostic use-wear of short-duration but repeated (distribution of the wear and intentional retouch of the edges indicates re-sharpening of the tool) non-woody plant processing together with an abrasive material was identified on both edges of the largest blade from Mine 83 (M-83-9) (Fig 6). Clear use-wear was also identified on both edges of the short curved blade from Mine 85 (M85-32), again, of repeated soft-plant processing with an abrasive substance/material. The use-wear identified on both blades is compatible with cereal processing, which was harvested close to the soil (the abrasive identified) to recover not only the cereal spike ears but the stems as well [52]. The rest of the blades made with Barremian-Bedoulian flint, whether retouched or not, bear no evidence of functional traces even though the possibility that they may have been used sporadically cannot be totally discarded. The blade made with white medium-grained flint (M84-171) did not display any diagnostic use-wear, nor did the two trapezes and arrowheads found in Mines 83 and 85 respectively.

### 3.2. Mines 5/11 and 16

**Fig 7:**
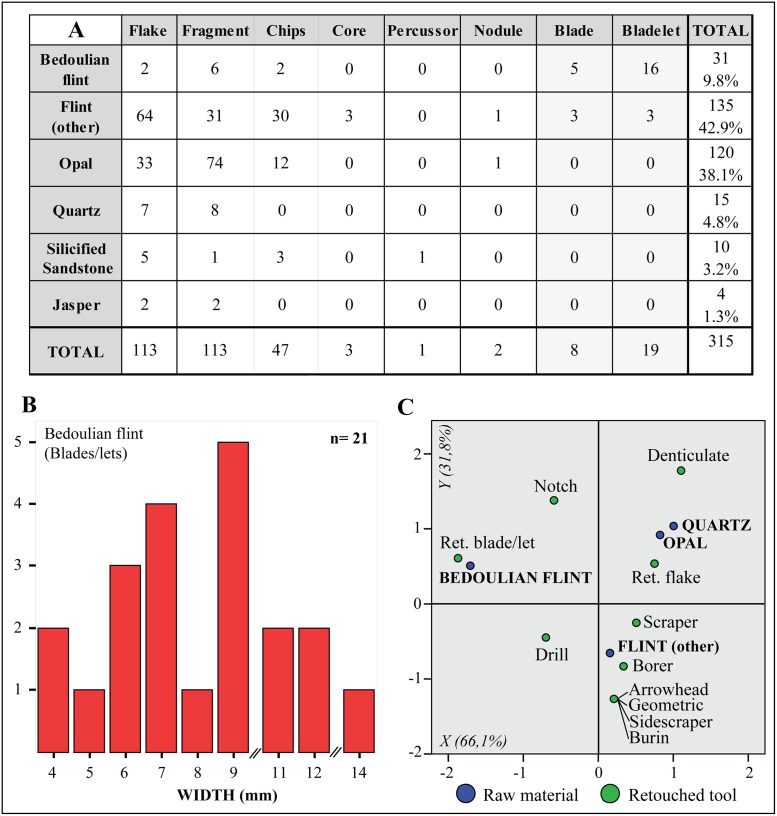
General breakdown of the lithic assemblage by raw materials and main technological categories (A); width of the blades and bladelets made with Barremian- flint (B); and correspondence analysis of the whole assemblage showing the variable raw material and retouched tool type (C).

The lithic assemblage from Mines 16 and 5/11 totals 315 artefacts. Non-Barremian-Bedoulian flint and opal are by far the most abundant raw materials in the fill of the mines (Fig 7). Barremian-Bedoulian flint was relatively abundant, accounting for almost 10% of the assemblage, while quartz, silicified sandstone and jasper were scarcely or very scarcely used. The diversity of raw materials is thus noteworthy, as well as their provenance, from strictly local to exogenic. Opal nodules were procured at the mines themselves, as layers of small- to medium-sized nodules are often found embedded in the Silurian grey shales the mines were partly excavated in [27]. Jasper, a raw material intensively exploited during the Early Neolithic in the Barcelona plain [53], was most probably procured from primary outcrops located on the hill of Montjuïc (Barcelona), located just 13 km from Gavà as the crow flies, as well as the few artefacts made of silicified sandstone, which is often found in association (in the same blocks) with jasper from Montjuïc. Flint types other than Barremian-Bedoulian constitute a heterogeneous group of fine to medium-grained versicolour varieties (translucent pink, white, dark brown), commonly found in prehistoric sites around the Barcelona plain and which have traditionally been considered as local raw materials although specific studies are needed to confirm that. Barremian-Bedoulian flint is thus the only non-local/exogenic raw material found in the fill of the Neolithic mines studied here.

**Fig 8:**
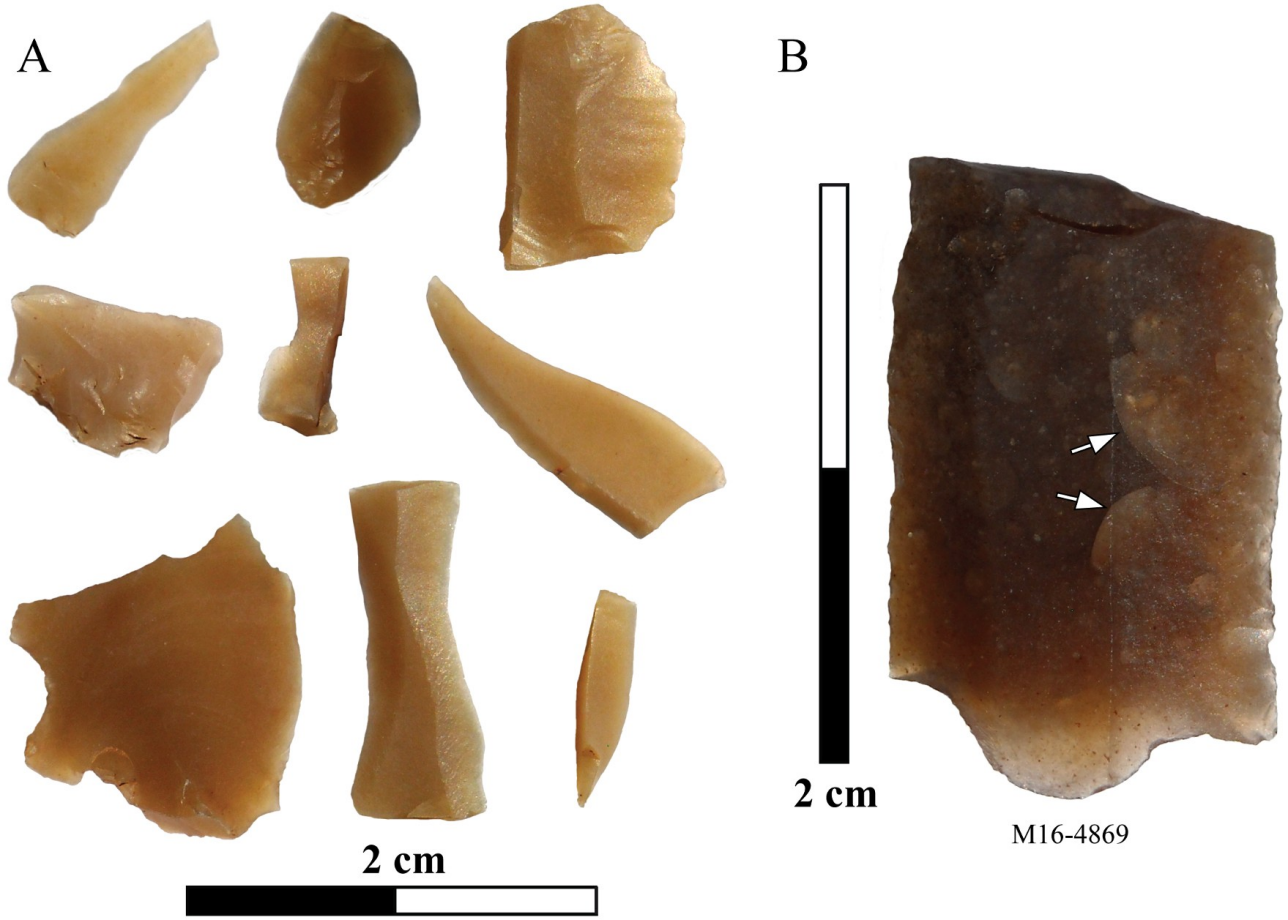
Small fragments and chipped debris of Barremian-Bedoulian flint from Mine 16 (A) and macroscopic scales on a thermally altered surface of a blade fragment in Barremian-Bedoulian flint (B).

**Fig 9:**
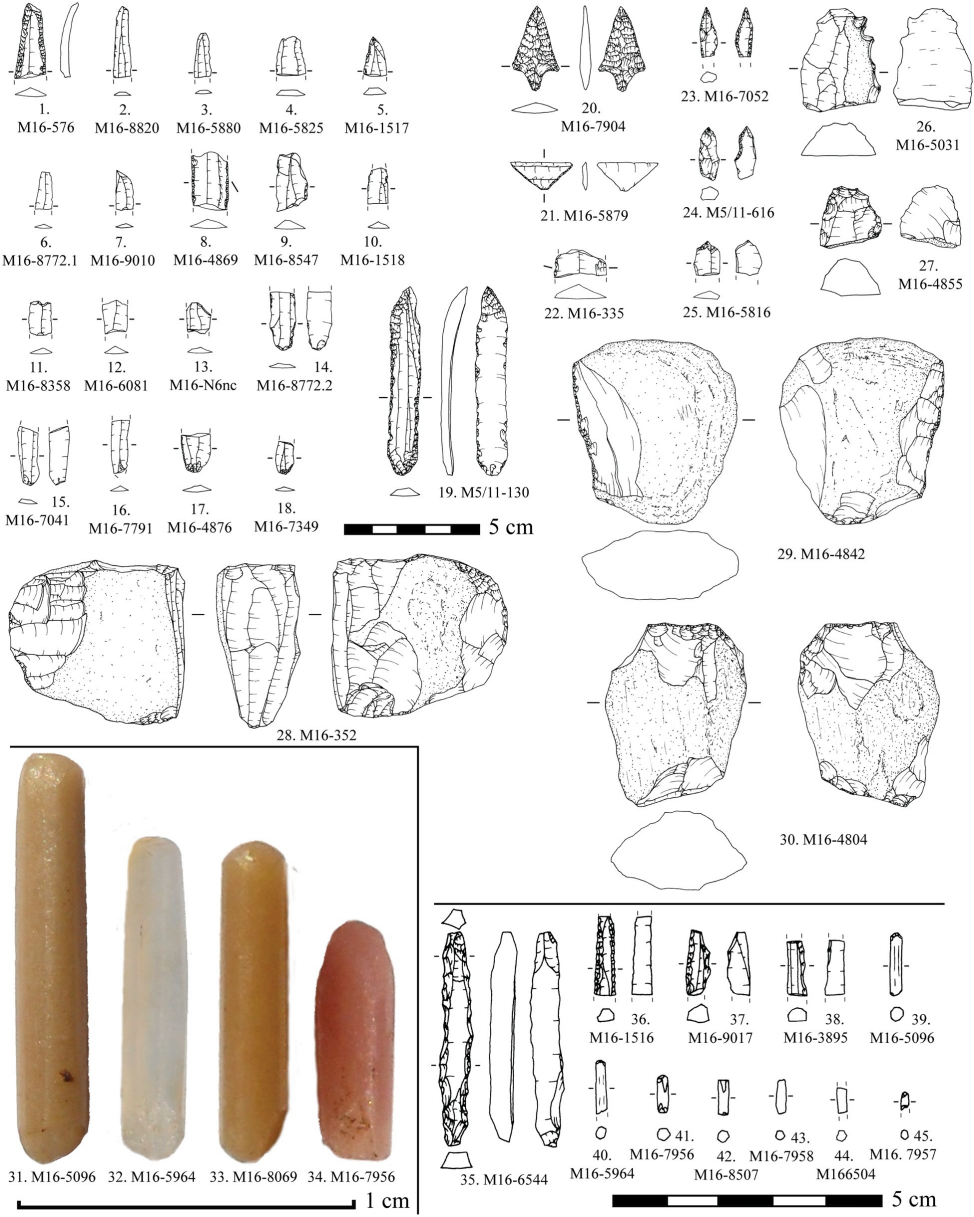
Blades and bladelet fragments made of Barremian-Bedoulian flint (n° 1–16 and 18; n° 17 is burnt); a blade core of non-Barremian-Bedoulian flint (28) and some of the retouched tools studied (n°. 19, 31, 33, 36, 37, 39 and 45 correspond to Barremian-Bedoulian flint; n°. 20–25, 27, 28, 32, 34, 35, 38 and 40–44 to non-Barremian-Bedoulian flint; and n°. 26, 29 and 30 to opal.

The Barremian-Bedoulian assemblage is dominated by flakes, fragments and small chipped debris, while blades (width ≥ 10 mm) and bladelets (width < 10 mm), mostly fragments as only one blade is complete, account for little more than 8% of the total. A breakdown of the lithic assemblage reveals a patterned use of the different raw materials identified and the coexistence of a series of *chaînes opératoires* at Gavà to produce flakes using opal, local flint types and, in lesser percentages, quartz, jasper and silicified sandstone; blades (unidirectional) by direct percussion made with relatively fine-grained local flint varieties; and, finally, small blades and bladelets using Barremian-Bedoulian flint (Figs 7, 8 and 9). The technological and morphological features of these blades and bladelets suggest, despite being fragmentary, the use of the pressure technique to produce the laminar blanks made of Barremian-Bedoulian flint. In this case, comparison of the width values, ranging between 4 and 14 mm, with experimental studies [54], reveals the coexistence of at least two modes of pressure technique, long crutch standing (mode 4) for the small blades and, secondly, short crutch sitting (mode 3) and/or shoulder crutch (mode 2) for the smaller bladelets (Fig 7B). Interestingly, the imported Provençal flint is not only represented by laminar blanks but also by a few flakes, chips and indeterminate fragments, some of which (as occurs with a couple of the laminar blanks) display post-depositional thermal alterations (Fig 8B).

The number of retouched tools is relatively high (54), representing 17.3% of the total lithic assemblage. Most of these were made with non-imported flint (29), opal (14) and Barremian-Bedoulian flint (10), while quartz (1) was very rarely used. The range of retouched tools represented is varied, dominated by retouched flakes (17, 31.5%), drills (11, 20.4%), borers (7, 13%), retouched blade/lets (6, 11.1%), scrapers (6, 11.1%) and, in smaller percentages, notches (2, 3.7%), arrowheads (1, 1.9%), geometrics (1, 1.9%), sidescrapers (1, 1.9%), burins (1, 1.9%), and denticulates (1, 1.9%) (Fig 7C). The high frequency of drills and borers is noteworthy, and associated with variscite bead production tasks, which might have taken place not far from the mines. Opal (and quartz) were used for the production of relatively large denticulates, scrapers and retouched flakes, whereas non-imported flint was used for a range of tools made of both flakes and blades, including projectiles, burins, drills, borers, etc. Interestingly, Barremian-Bedoulian flint bladelets were used not only as cutting tools, with little modification of the edges, but they were also transformed into small drills for bead-piercing (Fig 9).

**Fig 10:**
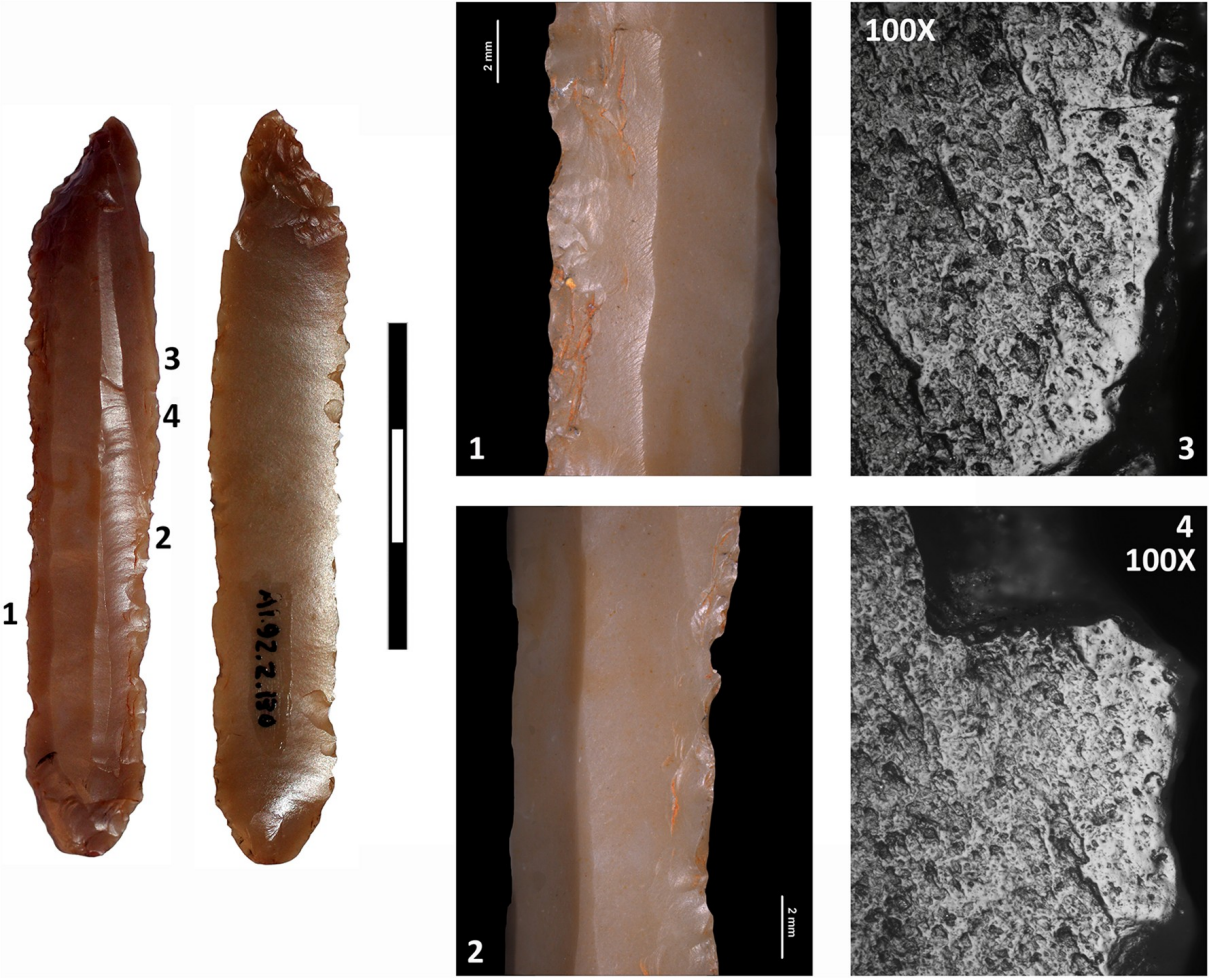
Small blade of imported Provençal flint (M5/11-130) showing both edges used for indeterminate soft plant cutting. Use-wear is seen microscopically on both retouched edges (1–2). The characteristics are very similar in the two cases: rounded polish with a significant degree of compaction (3–4). It must therefore have been used to cut soft plant matter, as the polish extends towards the centre of the artefact.

**Fig 11:**
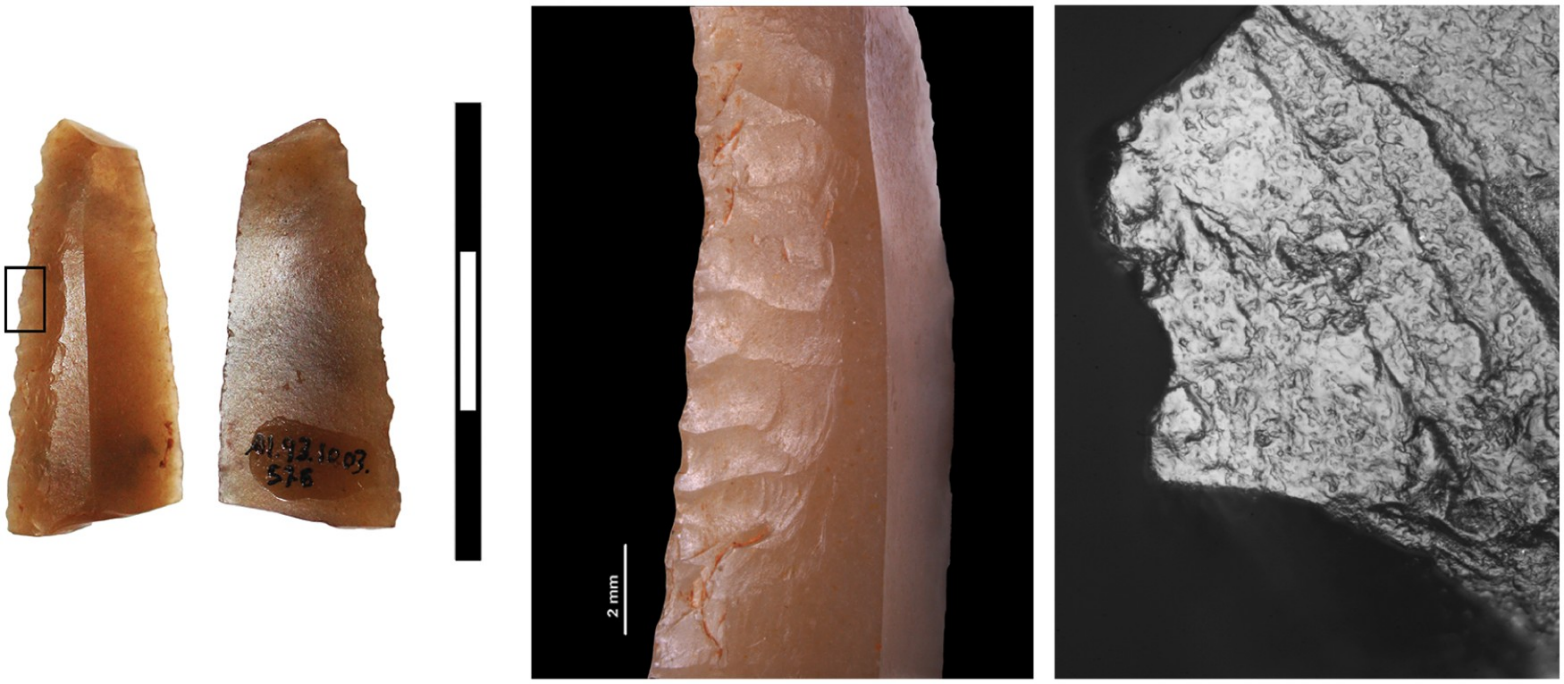
Honey flint blade (M16-576), retouched on both faces and used to cut plants. Very light plant polish (convex appearance and not very compact) is seen microscopically on the right edge. This appearance is probably due to it being used for a short time and the resharpening of the edge, which removed part of the active zone.

**Fig 12:**
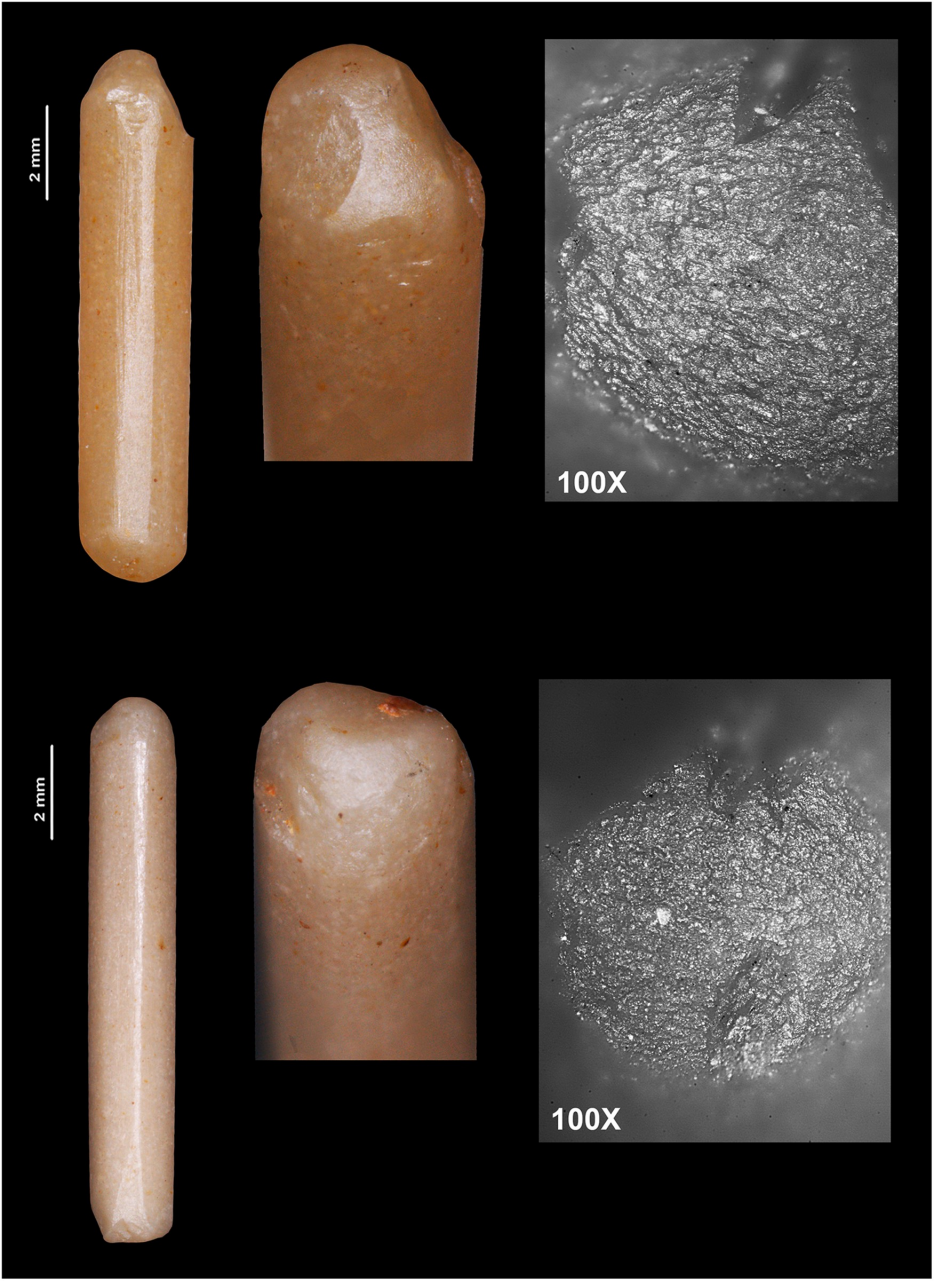
Drills made with honey flint bladelets. The upper one (M16-8069) was used at both ends, which have become totally rounded. The macroscopic photograph shows scars that continued to be rounded. This is probably the result of the pressure exercised during the work. The lower drill (M16-5096) was also used at both ends. Some scars produced during the drilling work can be observed at the distal end, while the proximal end seems to have broken but continued in use.

A total of 281 lithic artefacts were selected for use-wear analysis, following the previously-specified methodology/procedures. In most cases (191), the analysed artefacts displayed no functional polish, microscopic damage or chipping, thus indicating they had not been used. Some of these objects are probably knapping waste or, in the case of opal, waste products generated during the excavation of the mine. In addition, a group of 45 could not be analysed due to different alterations (patina, thermal alterations, etc.) on the edges. Finally, the analysis succeeded in identifying diagnostic use-wear on 44 artefacts, including 17 made of Barremian-Bedoulian flint (Table 1 and Figs 10–12).

**Table 1:** General breakdown of the lithic artefacts displaying diagnostic use-wear.

		BARREMIAN-BEDOULIAN FLINT		FLINT (OTHER)		OPAL	
		Flake	Blade/let	Flake	Blade/let	Flake	TOTAL
**Projectiles**	*Hunting/Warfare*	-	-	-	**2**	-	**2**
**Animal processing**	*Skinning/De-fleshing*		**1**			**1**	**2**
	*Hide working (cut*, *scrape & bore)*	-	**1**	**2**	**2**	-	**5**
**Plant processing**	*Non-woody plant processing (cereal cut)*	-	**3**	-	**1**	-	**4**
	*Non-woody plant processing (indet*. *cut)*	-	**2**	**1**	**2**	-	**5**
	*Wood processing*	-	-	**2**	-	**3**	**5**
	*Indeterminate*	-	**1**	-	**1**	-	**2**
**Stone working**	*Scrape*, *incise & bore*	**1**	-	**3**	**1**	-	**5**
	*Drill*	-	**4**	-	**2**	-	**6**
**Indeterminate (soft)**	*Cut*	-	**3**	-	**1**	**2**	**6**
	*Bore*	-	-	**1**	-	-	**1**
**Indeterminate (medium-hard)**	*Scrape*	-	-	-	-	**1**	**1**
	**TOTAL**	**16**		**21**		**7**	**44**

Results of the use-wear analysis confirm that Barremian-Bedoulian flint was used for a range of tasks, including plant processing, animal processing and stone working, in the same way as other flint types were used. Concerning stone working, Provençal flint bladelets were the preferred blank, though not exclusively, to make drills (Figs 9: 31–34 and 12) that were subsequently used for variscite bead piercing. This is indicated by the marked rounding of the active zones of the drills, associated with micropolish that is not very compact and with little evidence of abrasion. This indicates that the drilled substance was a soft and non-abrasive rock, such as variscite.

Results also confirm that opal, which was procured as a secondary product through variscite mining, was exploited and used in different tasks.

### 3.3. Heat-treated or not?

All artefacts made of imported Barremian-Bedoulian flint from Mines 83, 84, 85, 5/11 and 16 were closely inspected for macroscopic stigmata that would be diagnostic of heat-treatment, as heat treatment has previously been described for Neolithic production sites in the French Vaucluse region. This is particularly the case for bladelet production between the end of the 5^th^ and beginning of 4^th^ millennia cal. BC (e.g., [12,34,55]). However, in the assemblage from Gavà, despite the good preservation of most of the material, with many complete blades and some cores, no unequivocal evidence of heat-treatment (the presence of matt pre- and shiny post-heating removal scars on a single piece) was identified. In some cases, we suspected heat-treatment because of slight differences in the overall gloss intensity between different blanks. This aspect, together with the presence of slight changes in the colour of some of the cores, suggested that heat treatment might have been involved in the preparation of the raw materials before starting full production of blades/bladelets. In light of the difficulties in being conclusive in this respect, a sample of 12 artefacts were analysed using infrared spectroscopy and following previous methodology for identification of heat treatment in Barremian-Bedoulian flint [34]. The assemblage included different types of blanks, such as 2 complete large blades (M83-9, M84-50), two complete small blades (M83-13 [refitting the core in Fig 4], and M5/11-130), one fragment of a blade (M16-576), 5 fragments of bladelets (M16-1518, M16-7041, M16-8772.2, M16-8820, M16-9010) and two fragments of a blade and of a bladelet [M16-4869, M16-6081]) displaying micro-cracks and incipient pot-lid fractures (Fig 8B).

The methodology used is described in detail in Schmidt *et al*. [34] and only the details absolutely necessary for understanding our results are summarised here (all parameters for the measurements were also taken from [34]): Schmidt *et al*.’s technique aims to find pore-closure induced by heating of chert and flint artefacts. It is entirely non-destructive, as infrared spectroscopic measurements are conducted by transmission though the thin parts of artefacts. The result is a ratio (called hydration ratio), calculated from the infrared spectra, expressing the quantity of pore-space in the samples. If high compared to an unheated reference, the piece was subjected to heat at least once in its history. The absolute value of the hydration ratio can be correlated to heating temperature if an experimental reference collection is used for comparison.

**Fig 13:**
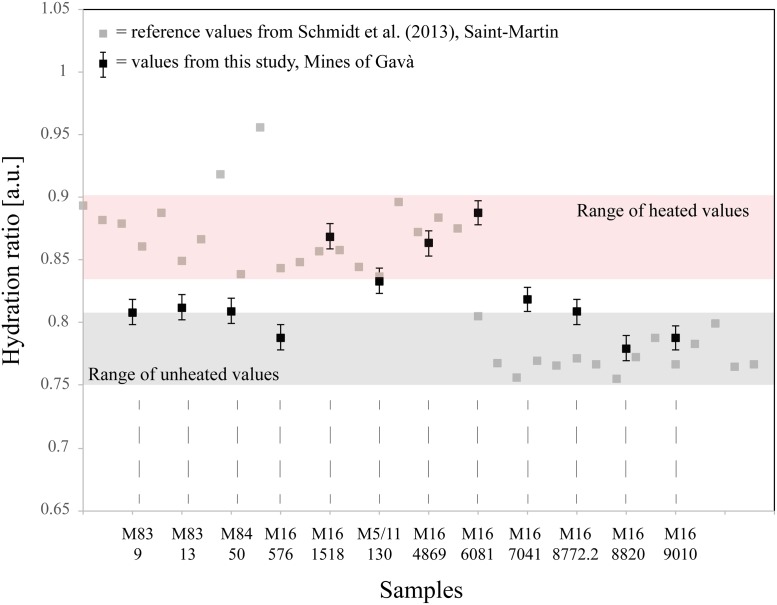
Results of the infrared analyses aiming at determining whether the Minas de Gavà pieces were heat-treated. Black dots with error bars are the samples from this study and grey dots are values from Schmidt *et al*. [34] obtained on heated and unheated pieces of the same Vauclusian flint for comparison. Note that four samples plot in the range of heated reference values, revealing that they were heated to similar temperatures.

Comparison of the ratio values obtained from the 12 analysed pieces from the Gavà Mines with the heated and unheated reference samples from the French production site of Saint-Martin (e.g., both assemblages did, *a priori*, originate in the same Lower Aptian outcrops located in the Vaucluse region) shows that 8 artefacts from Gavà plot in the same range as the unheated Saint-Martin reference pieces if measurement errors are taken into account, and 4 pieces plot in the range of the heat-treated reference (Fig 13). This result indicates that four of the pieces were heated (post-depositionally or intentionally) and 8 pieces have never been subjected to heat during their life cycle. It is likely, however, that the four pieces were intentionally heated in controlled conditions because their absolute ratio values between 0.83±0.01 and 0.89±0.01 correspond to heating temperatures between 200°C and 250°C for this flint from the Vaucluse (if compared to the data from [34]). Post-depositional burning is not likely to produce such temperatures but temperatures close to 250°C have previously been described for intentional heat treatment in the Neolithic *Chasséen* culture [35].

## 4. Discussion: The status of Barremian-Bedoulian flint in north-east Iberia during the Middle Neolithic

Barremian-Bedoulian flints from the Vaucluse were distributed outside the *Chasséen* cultural area over a wide region of north-eastern Iberia during the Middle Neolithic (ca. 4200–3600 cal. BC), penetrating the *Sepulcros de Fosa* culture sphere. This region, which *grosso modo* corresponds to modern Catalonia, extended from the southern Pyrenees to the Ebro River estuary and constituted the south-westernmost end of the distribution networks of Provençal flints. In this region, which was not part of the massive diffusion area, imported Barremian-Bedoulian flint (cores, finished blanks and finished tools) is fairly predominant in the abundant burial contexts dated in that period, indicating a clear shift in the status of Barremian-Bedoulian lithic industries for other cultures at very remote consuming sites. However, the status, use and management of Barremian-Bedoulian flints in Catalonia was far from being that simple, as old assemblages are being re-studied and new sites (and lithic assemblages) discovered, excavated and studied.

### 4.1. The core area of the Barremian-Bedoulian flint distribution south of the Pyrenees

**Fig 14:**
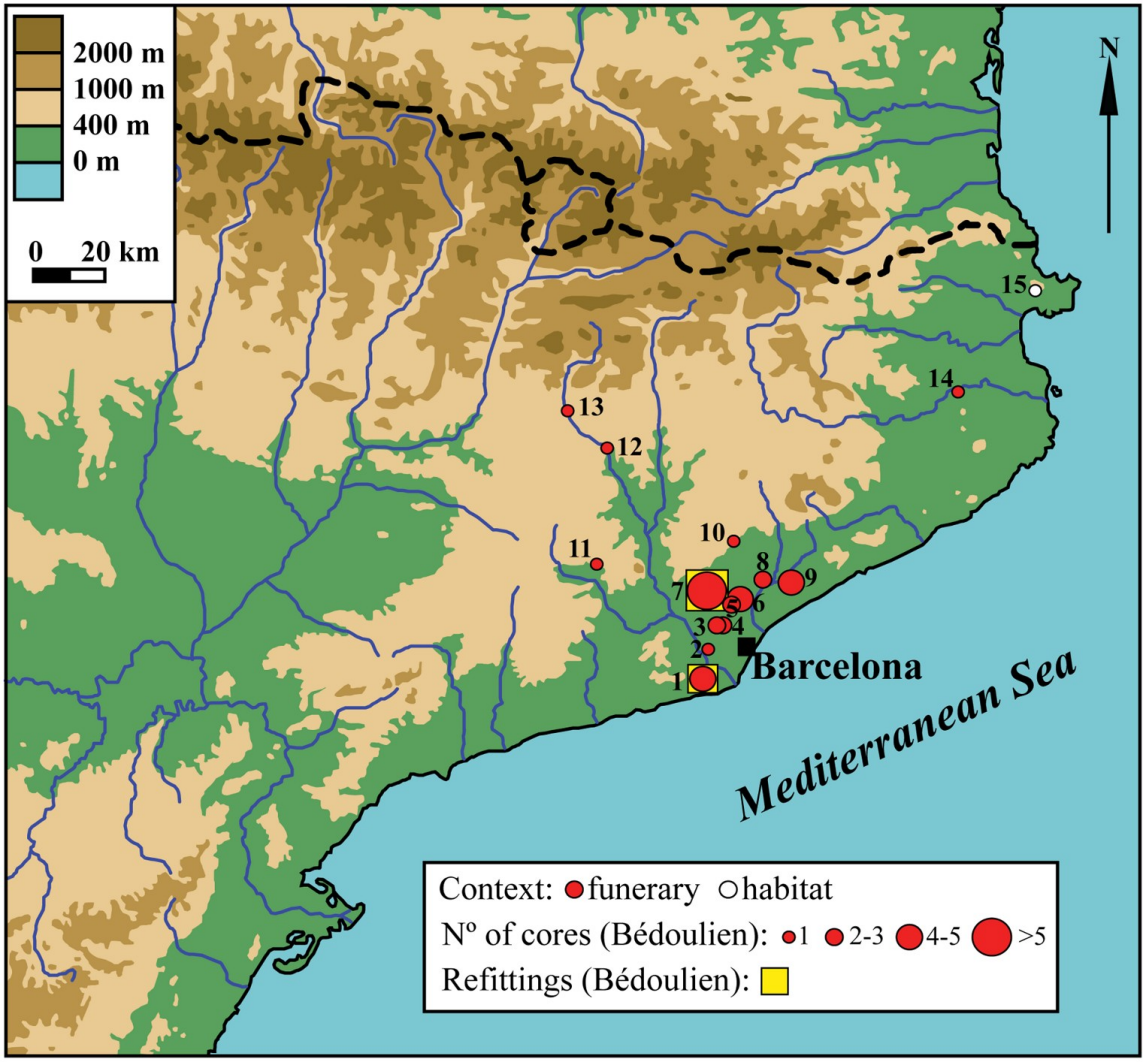
Spatial distribution of the 73 cores of Barremian-Bedoulian flint found south of the Pyrenees during the Middle Neolithic. 1: Mines de Gavà (n = 5), 2: Bòbila de Sant Joan d’Espí (n = 1), 3: Can Marcet (n = 3), 4: Can Fatjó dels Aurons (n = 1), 5: Bòbila Sallent (n = 2), 6: Bòbila Padró (n = 4–5), 7: Bòbila Madurell/Can Gambús I (n = 23+19), 8: Bòbila Bellsolà (n = 2), 9: Bòbila d’en Joca (n = 4), 10: Bòbila Negrell (n = 1), 11: Can Muset (n = 1), 12: Palà de Coma (n = 1), 13: Santa Constança (n = 1), 14: Sant Julià de Ramis (n = 1), 15: Ca n’Isach (n = 1).

During the later stages of the Early Neolithic (Neolithic Postcardial), thus contemporary to the early stages of the *Chasséen* culture, the presence of imported Barremian-Bedoulian flint south of the Pyrenees was scarce and had little impact in the local flake-dominated lithic industries with a small percentage of non-standardized blade components. The expansion in the diffusion of Provençal flints took place between the last quarter of the 5^th^ millennium and the first half of the 4^th^ millennium cal. BC and it represented a clear shift in the lithic industries of the period, a change also observed in other aspects of the material culture record, for instance, in funerary practices [56]. The flux of exogenic flints south of the Pyrenees was, however, far from homogeneous, geographically, in the numbers, or in the blanks that were distributed. Most of the Barremian-Bedoulian flint has been found at sites in a restricted region of the *Sepulcros de Fosa* culture located north-northwest of Barcelona, which corresponds to the Vallés Oriental and Vallés Occidental districts (e.g., Bòbila d’en Joca, Bòbila Madurell-Can Gambús I, Bòbila Negrell, Bòbila Padró-Can Tiana, Can Fatjó del Aurons and Can Marcet). It is in this small region of Catalonia where almost all the Barremian-Bedoulian cores (62 out of 73) are concentrated and, secondly, where refittings have been identified (eight bladelets at Bòbila Madurell [20]; three small blades and, finally, one blade with a core at Can Gambús I [57,58]), thus suggesting that the distribution of Barremian-Bedoulian flints south of the Pyrenees could have been controlled or modulated by certain groups of local elites from these sites, Bòbila Madurell in particular [20]. New evidence from Gavà led us to expand the core area of diffusion of Barremian-Bedoulian flint southwest of Barcelona, including the Baix Llobregat district (Fig 14). This newly-delimited area of optimal access to Provençal flints also corresponds to the centre of production and of maximum diffusion and use of variscite ornaments in Catalonia, thus indicating a strong connection between the import of Barremian-Bedoulian flints and the production and early diffusion and use of variscite from Gavà at the heart of the *Sepulcros de Fosa* cultural area. In addition, the total overlapping of the imported Provençal flint and variscite ‘core’ areas confirm that variscite from Gavà was one factor, if not the principal one, attracting Barremian-Bedoulian flint towards the centre of the *Sepulcros de Fosa* cultural area in north-eastern Iberia, from where part of it was subsequently redistributed to other regions. In this respect, there is little doubt that the Neolithic variscite mines at Gavà were a key nodule in the complex exchange networks operating around the western Mediterranean basin and, accordingly, a centre attracting exogenic materials (Provençal flint, obsidian, Alpine rocks, etc.), products, and new ideas and knowledge from distant regions such as southern France and northern Italy.

### 4.2. The status of imported Barremian-Bedoulian flint in north-eastern Iberia

Results of the comprehensive approach to the lithic assemblages from the variscite mines at Gavà provide irrefutable evidence of the triple status (functional, symbolic and both) of Barremian-Bedoulian flint within the community of miners, as previously observed at Bòbila Madurell-Can Gambús I [20,57], revealing unexpected complexity in the modes of consumption and use of Provençal flint at the heart of the *Sepulcros de Fosa* culture area.

**Fig 15:**
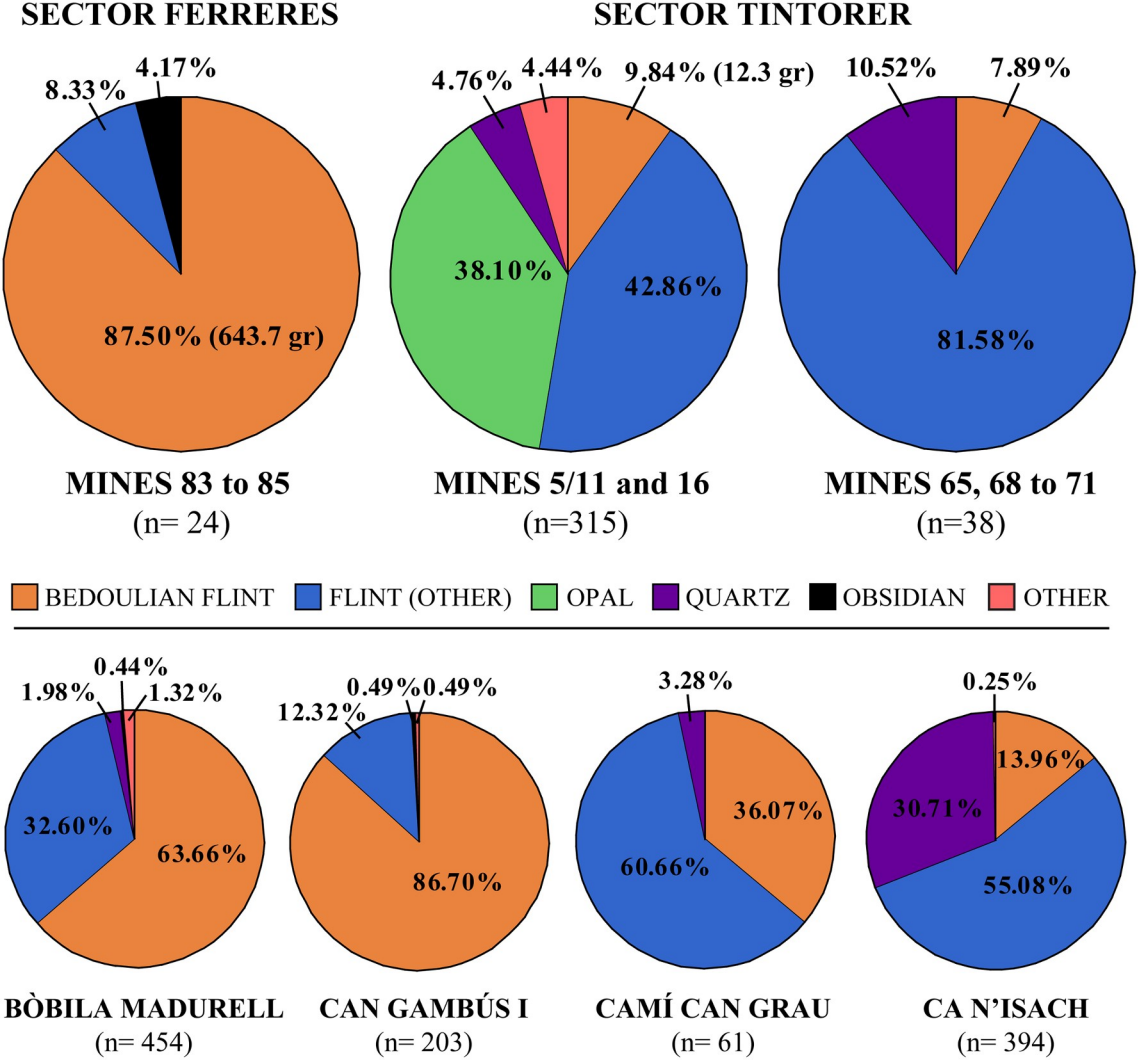
Breakdown by raw materials of the lithic assemblages from a series of contemporary Middle Neolithic contexts in Catalonia (data from refs. [20,30,58]).

Imported Barremian-Bedoulian flints were frequent and predominant in the abundant Middle Neolithic funerary record in Catalonia. Almost all the cores (except for a very small core found at the open-air settlement of Ca n’Isach) and of the larger and complete blade/lets and finished products (projectiles) have been recovered from funerary contexts, thus indicating that most of the imported industries were preferred for symbolic purposes. However, these precious and highly valued goods were not always produced *ex professo* to be deposited in the graves but often had a previous history of use behind them, probably within the same community. In addition, other flint types, often local, were also used to produce blanks and tools that were to be deposited accompanying the imported Barremian-Bedoulian artefacts and the rest of the grave goods (e.g., axes, ceramic containers, variscite ornaments, etc.). At the same time, as shown by their recurrent presence in Middle Neolithic non-funerary contexts at Gavà, Bòbila Madurell-Can Gambús I, Ca n’Isach and Serra del Mas Bonet (Fig 15), Barremian-Bedoulian productions were also integrated into local, mostly flake-dominated, lithic industries. Imported flints were used, though in smaller numbers, to produce a wide variety of tools and participated in tasks as diverse as hide-working, soft plant and wood-processing and stone-working.

In conclusion, Barremian-Bedoulian industries found in contexts belonging to the *Sepulcros de Fosa* culture sphere were both appreciated and used as everyday tools and, to a higher degree but not exclusively, as powerfully symbolic and valued goods [4,57]. Imported Provençal flint had thus a potent symbolic component, probably socially enhanced, though not *per se* but as artefacts (finished tools) or potential artefacts (complete blade/lets and non-exhausted cores) as indicated by the total absence of Barremian-Bedoulian flint flakes, fragments and chips from the abundant funerary contexts. A phenomenon that, on the contrary, indeed occurred with variscite, as broken beads and small and roughly-faceted fragments of variscite have been found as part of the grave goods in the funerary contexts at Gavà [5], thus indicating a stronger symbolic value of variscite, of which even the smallest fragments were highly valued.

### 4.3. Diversity of Barremian-Bedoulian industries at the consuming sites and producing centres

During the first quarter of the 4^th^ millennium cal. BC, a wide range of Barremian-Bedoulian flint blanks and cores were being used by the community of miners at Gavà in the core of the *Sepulcros de Fosa* culture area. Imported flints found south of the Pyrenees included complete large blades (length ≥ 10 cm, very rare: 4 at Gavà, 3 at Bòbila Madurell and 1 at Camí de Can Grau), small/short blades (length < 10 cm, width ≥ 1 cm), bladelets (width < 1 cm) and cores (n = 73), mostly non-exhausted medium to small sized blade and bladelet cores. For the larger/wider laminar blanks, the standing pressure technique (mode 4) was used, while the small bladelets might have been detached by the short crutch sitting pressure technique (mode 3), thus indicating that two modes coexisted. In addition, results of the heat-treatment study show that the larger blades were not produced from heated cores and that the small blades and bladelets were sometimes heated and sometimes not. Concerning the cores, it is noteworthy that one of the non-heated small blades refitted with one of the cores (Fig 5A), the one which is also associated with the other three refitted blades (Fig 5B), thus indicating that at least some unheated blade cores circulated to the final extreme of the exchange networks.

In view of these results, although some aspects are to be refined in further work (e.g., the extent of heat-treatment within Barremian-Bedoulian industries), it can be concluded that imported Provençal industries found in north-eastern Iberia during the first half of the 4^th^ millennium were diverse. First, they were formed by a range of quite different products (large blades, short blades, bladelets and non-exhausted cores). Secondly, different modes/variants of pressure technique were used to detach laminar blanks, from large blades to small bladelets. Finally, heat treatment was a common technical choice in order to improve the knappability of Barremian-Bedoulian flints. Its use, however, might have not been as widespread or systematic as hitherto proposed and certainly was not a rule nor the response to a technical constraint or the poor quality of the raw material, thus indicating that it was a cultural choice or trait. In conclusion, the study of Barremian-Bedoulian industries found at the distant consuming sites located south of the Pyrenees reveals different *chaines opératoires* involved in the production of Barremian-Bedoulian industries and that production processes were far from being homogeneous. Therefore, assuming that Barremian-Bedoulian industries found in NE Iberia were mostly produced in Provence (see the discussion in the next section), it is reasonable to advocate that this diversity is reflecting certain heterogeneity in the production processes of Barremian-Bedoulian industries at the Provençal producing centres (whether at the specialized workshops in the immediate vicinity of primary outcrops or at the nearby consumer-redistributor sites located at less than one-day walking distance). An interpretation that, to some extent, might be in certain disagreement with interpretations stressing the technological stability and qualitative standardization of the Barremian-Bedoulian industries found/produced at the Vauclusian sites and, by extension, the French Midi during the *Chasséen* [9–11].

### 4.4. Who knapped the imported Barremian-Bedoulian flints found south of the Pyrenees?

The presence, though in relatively small numbers, of small flakes, chips and mundane tools such as scrapers and borers made of Barremian-Bedoulian flint in the few non-funerary Middle Neolithic contexts/sites found south of the Pyrenees (e.g., Gavà Mines, Bòbila Madurell, Serra del Mas Bonet, Ca n’Isach) leaves no doubt that imported flints were, at least to a certain degree, knapped *in situ* by local groups to produce a range of non-specialized tools for everyday tasks (e.g., scrapers, borers, sickle blades, etc.). However, determining who produced the complete laminar blanks, found almost exclusively in the funerary contexts, is still debated. Three main hypotheses can be summarized: 1) The laminar blanks and cores found south of the Pyrenees were imported as finished products, so the blades were never produced *in situ*; 2) The laminar blanks were fully or partly knapped *in situ* by itinerant specialized knappers from neighbouring regions north of the Pyrenees; or 3) Some members belonging to the local Neolithic communities (specialized knappers?) had acquired the expertise to knap by pressure at least part of the laminar industries made of Barremian-Bedoulian flint, which they produced using the non-exhausted cores that were imported to the region. The scarce and sometimes contradictory evidence does not allow us to be fully conclusive in this respect, subsequently hindering a full understanding of the nature and functioning of the supply systems/exchange networks involved in the circulation of Vauclusian flints south of the Pyrenees. Despite this, recent data from Gavà and its contextualization within the *Sepulcros de Fosa* framework shed light on some key aspects of the diffusion systems.

It is highly unlikely that the longest blades (≥ 10 cm long) found south of the Pyrenees were produced *in situ*, either by itinerant or local knappers, as indicated by several factors. These were highly exceptional and rarely-found items that clearly stand out by their size from the rest of the laminar products found in the region. In addition, there is not a single one of the non-exhausted Barremian-Bedoulian cores found in Catalonia that could remotely have produced laminar blanks of that size. In this sense, the measurements of the cores found correlate better with the size of the more frequent medium- to small-sized blades and bladelets from 6 to up to 8.5 cm long. In consequence, it seems reasonable to assume that, in concordance with previous interpretations [39], the longer blades were produced somewhere closer to the primary sources, most probably in Provençal *Chasséen* settlements or workshops and that some of the items produced were selected for exchange. However, what is still less clear is where and when those long blades which display retouch and/or micro use-wear were used: near the production centres? At the distant consuming sites? Or somewhere between the two?

**Fig 16:**
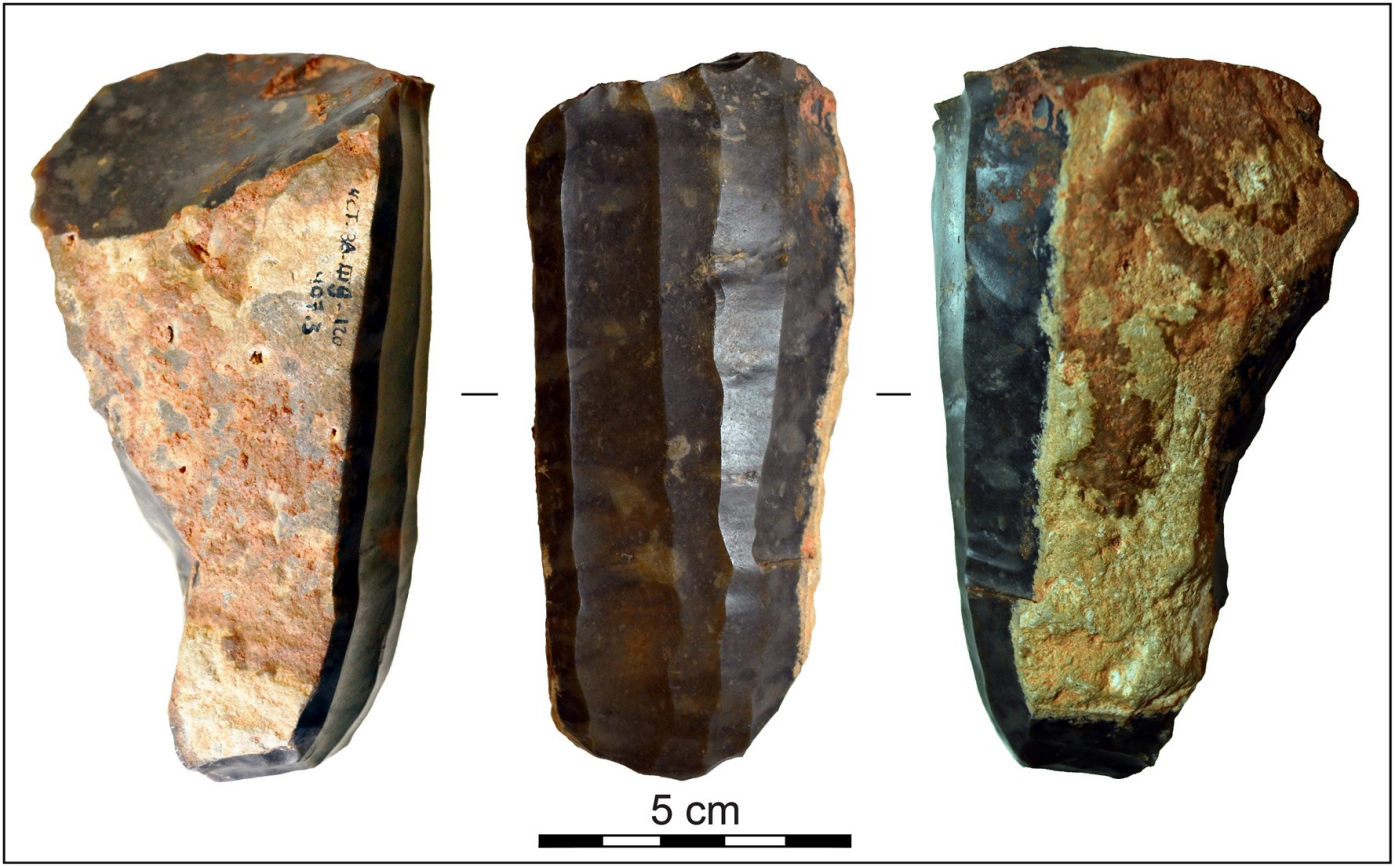
Blade core made of Lacustrine Oligocene banded flint found in Mine 3. The nearest outcrops known are in the Castelltallat Formation [61], almost 80 km north-west of Gavà.

Regarding the production of smaller blades and bladelets, the scenario is rather more complex. The presence of refits (blade-blade and core-blade) of non-used laminar blanks found at burial sites located at the core of the *Sepulcros de Fosa* culture area, where most non-exhausted cores are found too, supports the idea that at least part of the Barremian-Bedoulian blades knapped *ex professo* for funerary purposes might/could have been produced *in situ* south of the Pyrenees, whether by local or non-local knappers. It has also been proved that imported flints were also integrated in local industries for common uses by the end of the 4^th^ millennia cal BC. Those tools (scrapers, knives, burins, drills, etc.) were also produced *in situ*, in this case obviously by local communities. In view of this evidence, it seems reasonable to assume that at least part of the complete blades and bladelets found in funerary contexts which display retouch or microscopic use-wear might have been produced *in situ*, in the same way as the items produced *ex professo* as grave goods, and, therefore they would have been used by the local communities prior to their deposition in the funerary contexts. All in all, increasing evidence from the studies of Barremian-Bedoulian assemblages found at the end of the exchange networks seems to advocate the recurrent production of Bedoulian laminar products south of the Pyrenees. However, with the current data it is not possible to discriminate (except for the larger blades) between what was produced *in situ* by knapping the ready-to-use cores and what arrived as finished items, or to determine the social actors performing the *in situ* productions in north-eastern Iberia (local or itinerant knappers [specialists?] operating on both sides of the Pyrenees). This makes it difficult to know if the pressure technique did or did not diffuse to the core area of the *Sepulcros de Fosa* culture during the Middle Neolithic and to ascertain the degree to which social interaction established between the *Sepulcros de Fosa* and *Chasséen* culture spheres through the circulation of items and products also involved the exchange/diffusion of ideas and know-how between the two. In this sense, despite being an isolated case for this period, the find of a blade core made of banded dark-brown, fine-grained flint reduced by the pressure technique in a non-funerary context in Mine 3 (excavated in the late 1970s and tentatively attributed to the Middle Neolithic [59]) (Fig 16) is worth mentioning. On the one hand, it attests the use, albeit sporadic, of the pressure technique for laminar production during the Middle Neolithic using raw materials other than Provençal Barremian-Bedoulian flint. On the other hand, the flint used strongly resembles the dark-coloured banded flint found within limestone and marly limestone beds of Oligocene units in the northern part of the Ebro basin [60]. It suggests that this could represent an ‘adaptation’ of the *Chasséen* technique on a raw material from the Ebro basin and, consequently, supports the idea of a technical transfer of the pressure technique south of the Pyrenees. However, we consider that the current evidence is insufficient to prove such diffusion or to rule out the possibility that this core could have reached Gavà as a final product from a different techno-cultural sphere.

## 5. Conclusions

The integrated study of the lithic assemblages from the variscite mines of Gavà and its contextualization within the *Sepulcros de Fosa* culture sphere framework has revealed unexpected complexity in the modes of diffusion, consumption, use and status of Barremian-Bedoulian industries in north-eastern Iberia during the 5^th^ to 4^th^ millennia cal BC transition. In addition, the results provide complementary data to better understand relevant aspects of the nature and organisation of Barremian-Bedoulian flint exploitation and early supply systems at the Provençal producing-exporting sites themselves during the later phase of the *Chasséen* culture.

During the Middle Neolithic, most Barremian-Bedoulian flint that was distributed south of the Pyrenees became concentrated, as occurred with other exogenic materials such as obsidian and alpine rocks, in the core area of the *Sepulcros de Fosa* culture [4]. Local communities within this region, already controlling extraction, production and regional diffusion of variscite ornaments [5], seem to have also exerted control (or modulated) over the fluxes of Vauclusian flint towards this region. Accordingly, these communities also played a key role in its redistribution throughout, *grosso modo*, the modern region of Catalonia. Both phenomena constitute a significant indication of the status of those communities (Bòbila Madurell-Can Gambús 1, Gavà Mines, etc.) as both regional and trans-regional nodes of social interaction and exchange/redistribution. In this respect, and differently from what is proposed for the *Chasséen* culture area, settlement organization, articulation and hierarchization within the *Sepulcros de Fosa* culture sphere was not articulated by the use and status of Provençal industries or other exogenic materials, even though they undoubtedly played an important role in it.

Barremian-Bedoulian imported industries had, south of the Pyrenees, a triple status (functional, symbolic and both) as indicated by the presence of cores and complete non-used blades and bladelets produced *ex professo* in funerary contexts, complete used blades and bladelets in funerary contexts and, finally, of a range of utilitarian tools in occupational contexts. The interconnection between the symbolic and utilitarian status of Barremian-Bedoulian industries is also reinforced by the fact that most artefacts were found in funerary contexts (thus with a potent symbolic component) but always transformed into finished tools, potential tools (blades) or source of tools (non-exhausted cores), never as fragments or flakes.

If the status and ways of consumption of Barremian-Bedoulian industries at the heart of the *Sepulcros de Fosa* culture sphere were varied, so were the range of artefacts/products being used and consumed (large blades, blades, bladelets and cores), the techniques used (pressure modes 3 and 4, according to Pelegrin’s works [54]) and other technical procedures (some artefacts had been heat treated while others were not), thus indicating the coexistence, although maybe not in the same region, of several *chaînes opératoires* in the exploitation of Vauclusian flints. Reconstructing all the *chaînes opératoires*/production processes in order to determine what was knapped where, how and by whom through the limited and selected material that circulated to north-eastern Iberia is difficult if not impossible at the moment. However, our results have shown that some artefacts (large blades) were surely produced outside the *Sepulcros de Fosa* culture sphere, while part of the abundant blade component found at the numerous Middle Neolithic burials was produced within the region. In this latter case, the figure of itinerant specialists, working on both sides of the Pyrenees from redistribution centres, currently seems the most plausible hypothesis/interpretation to explain the *in situ* production of Barremian-Bedoulian flint artefacts but without pressure technique being widely diffused in the region. On the other hand, the presence of non heat-treated products (and cores) in north-eastern Iberia reveals that this technical procedure was not systematically used in the production of all Barremian-Bedoulian industries, subsequently indicating that its use was not a technical requirement or response to a constraint of the raw material or of the technique used (pressure) but mainly a cultural trait. Taken all together, this seems to indicate that the diffusion of Provençal flint through different cultural spheres involved not only changes in its status and forms of consumption but also certain de-standardization (increasing variability) in the production process/es of the artefacts found far from primary outcrops located in the Vaucluse region, as the non-exhausted cores circulating for hundreds of kilometres could be knapped by a wide range of knappers (from Provençal specialists to, maybe, itinerant knappers operating on both sides of the Pyrenees) with diverse abilities, varied needs and belonging to different cultural and lithic traditions, thus resulting in more varied productions at the far end of the circulation networks.
